# Infectious bronchitis virus: an overview of the “chicken coronavirus”

**DOI:** 10.1099/jmm.0.001828

**Published:** 2024-05-21

**Authors:** Marco Falchieri, Vivien J. Coward, Scott M. Reid, Tom Lewis, Ashley C. Banyard

**Affiliations:** 1 Avian Virology, Animal and Plant Health Agency (APHA)-Weybridge, Woodham Lane, Addlestone, KT15 3NB, UK; 2 WOAH/FAO International Reference Laboratory for Avian Influenza, Animal and Plant Health Agency (APHA-Weybridge), Woodham Lane, Addlestone, Surrey KT15 3NB, UK; 3 School of Biological Sciences, University of West Sussex, Falmer, West Sussex, UK

**Keywords:** avian coronavirus, IBV, poultry

## Abstract

Infectious bronchitis virus (IBV) is a highly contagious avian *Gammacoronavirus* that affects mainly chickens (*Gallus gallus*) but can circulate in other avian species. IBV constitutes a significant threat to the poultry industry, causing reduced egg yield, growth and mortality levels that can vary in impact. The virus can be transmitted horizontally by inhalation or direct/indirect contact with infected birds or contaminated fomites, vehicles, farm personnel and litter (Figure 1). The error-prone viral polymerase and recombination mechanisms mean diverse viral population results, with multiple genotypes, serotypes, pathotypes and protectotypes. This significantly complicates control and mitigation strategies based on vigilance in biosecurity and the deployment of vaccination.

## Graphical Abstract

IBV transmission pathway and viral electron microscopy image. The virus can spread between commercial farm either directly or indirectly through fomites, personnel or wild birds.

## Historical perspective

The first infectious bronchitis virus (IBV) detection was reported in the USA during the 1930s [1], with the virus being identified in 1936, following *in vivo* neutralization studies [2] and isolated in the USA in 1937 [3]. Early reports described the disease as a mild respiratory syndrome. In 1941, the first vaccine was developed. However, vaccination has been a contributor to the emergence of multiple diverse serotypes and pathotypes globally [4]. In 2018, it was decided that all bird-infecting gammacoronaviruses would be grouped into one species category. Among these, the variant previously identified as the IBV is the sole coronavirus that targets chickens [5].

## Clinical presentation

The upper respiratory tract is the primary site of virus replication, and the incubation period can vary between 18 to 36 h post-infection [4]. Following a transient viremia, the virus replication and dissemination in the host often results in infection of the oviduct, kidneys and caecal tonsils. The clinical picture can vary significantly depending on viral genotype, bird’s age, and nutritional and immunological status. IBV infections typically induce respiratory signs, such as gasping, rales, snicking, nasal discharge and watery eyes. Feed and water consumption might also be reduced. Clinical disease may be more evident in younger or unvaccinated chickens. Laying hens can have a reduction in egg production and decreased egg quality. Some strains are nephropathogenic and can severely affect kidney function. Importantly, IBV can trigger secondary bacterial infections, contributing to high mortality rates [4].

## Microbial characteristics: phenotypic and genotypic features

### Phenotypic characteristics

**Fig. 1.**
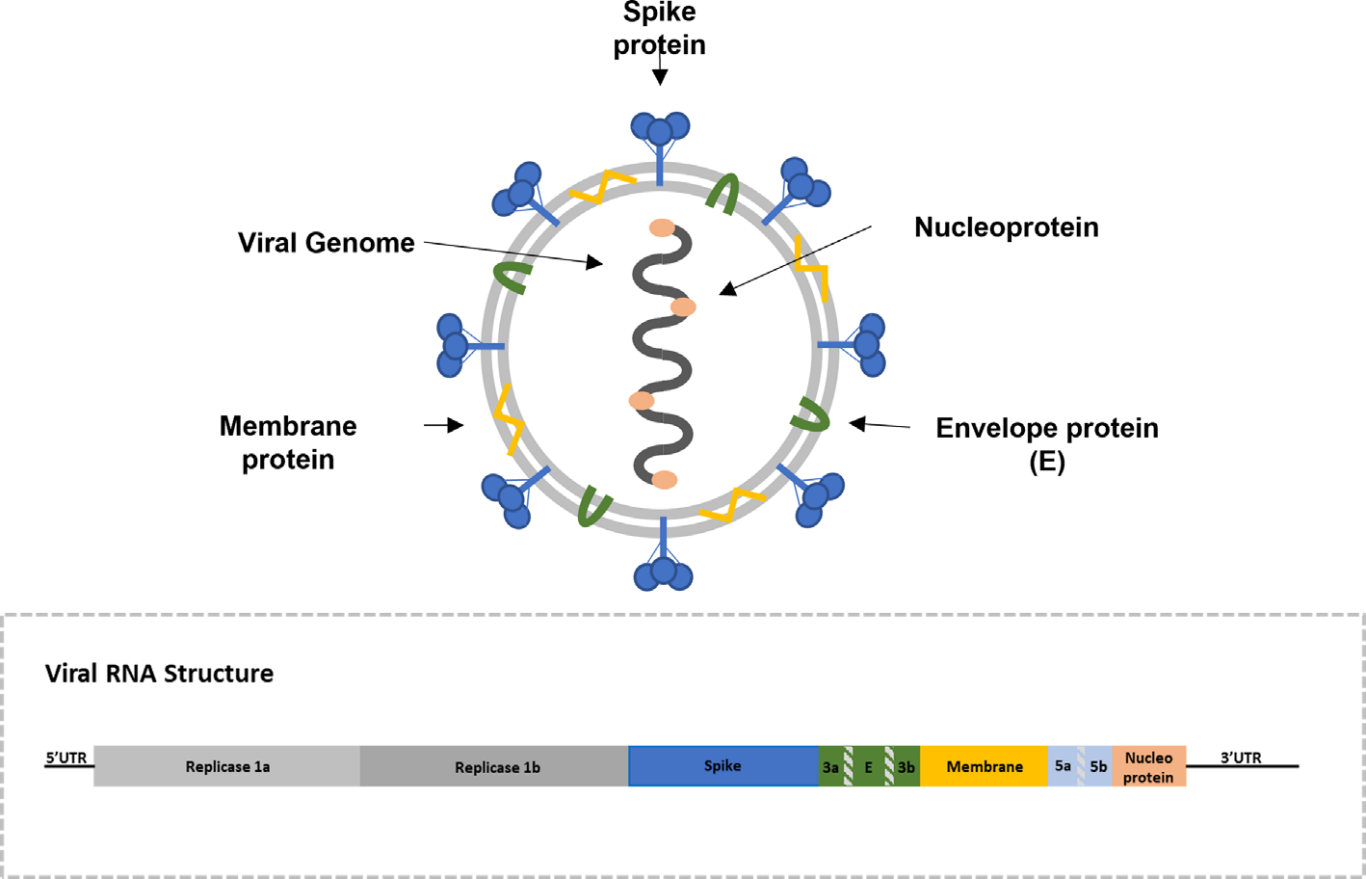
Schematic representation of the virus and its genome [6].

The IBV is an enveloped pleomorphic *Gammacoronavirus* within the *Coronaviridae* family. These viruses have a single-stranded positive-sense RNA genome of approximately 27.5–28 Kb. Virions are made up of spike (S), envelope (E), membrane (M) and nucleocapsid (N) structural proteins (Fig. 1). The S glycoprotein is involved in host-cell attachment, membrane fusion and cell entry. Viral isolates are classified antigenically or molecularly based on the Spike protein aminoacidic or nucleotide sequences [4].

The error-prone nature of the viral RNA-dependant RNA polymerase (RdRp) and the potential for viral recombination events are believed to have led to the emergence and evolution of multiple variants. However, some scientists have speculated that the former mechanism during replication is not expected to constitute a major role in the evolution of IBV, since RdRp possesses exoribonuclease activity that provides some proofreading errors during coronavirus replication [6].

Because of this genomic hypervariability, classification has historically been complicated and nebulous, and several methods have been suggested based on genotype, serotype, pathotype (based on organ tropism) and protectotype (classification of viral types by immunization and *in vivo* challenge). It is not known exactly how many IBV strains are present worldwide; however, a more recent classification based on the full Spike 1 subunit sequence has identified seven genotypes (GI-GVII), with dozens of genetic lineages [7].

Different serotypes generally have significant differences (20–50 %) in the deduced amino acid sequences of the S1 subunit. IBV serotypes that share more than 95 % amino acid identity in S1 should have cross-protection, whereas IBV strains of other serotypes that share less than 85 % amino acid identity do not cross-protect. Poor cross-protection has been described in viruses, clearly distinguishable in only 2–3 % differences in amino acid sequences [8]. The most prevalent strains in the UK are 793B (GI-13), Ma5 (GI-1) and QX (GI-19).

## Clinical diagnosis, laboratory confirmation and safety

### Clinical diagnosis

Clinical diagnosis is problematic as clinical signs or post-mortem findings of IBV are not pathognomonic, and multiple pathogens are considered during clinical evaluation, including pathogens such as Avian Influenza and Newcastle Disease virus. However, IBV should always be suspected when respiratory signs, a drop in egg production, and bacterial secondary infections are diagnosed (e.g., *Escherichia coli*) [9].

### Laboratory confirmation

Sample source: oropharyngeal and cloacal swabs, but also target organs/tissues (such as nasal turbinates, trachea, caecal tonsils and kidneys), are the most useful samples for confirmatory laboratory diagnosis. Virus isolation is commonly performed in embryonated chicken eggs and tracheal organ cultures [4].

Diagnosis relies on molecular [reverse transcription polymerase chain reaction (RT-PCR)] and serological assessment of submitted samples. Due to the widespread distribution, viral persistence in the host, and mass vaccine administration, subtyping analyses are required to define the infecting agent. This can include type-specific RT-PCRs or genotyping analysis. As several live vaccines are available commercially and viral persistence can last months, particularly in the digestive tract, the interpretation of diagnostic results can be challenging, and findings should be interpreted carefully based on vaccination history, viral distribution and clinical picture. Serological techniques, such as ELISA and haemagglutination inhibition assay (HIA), are commonly used to detect vaccine-derived naturally acquired antibody responses to IBV [4].

### Pathology

**Fig. 2.**
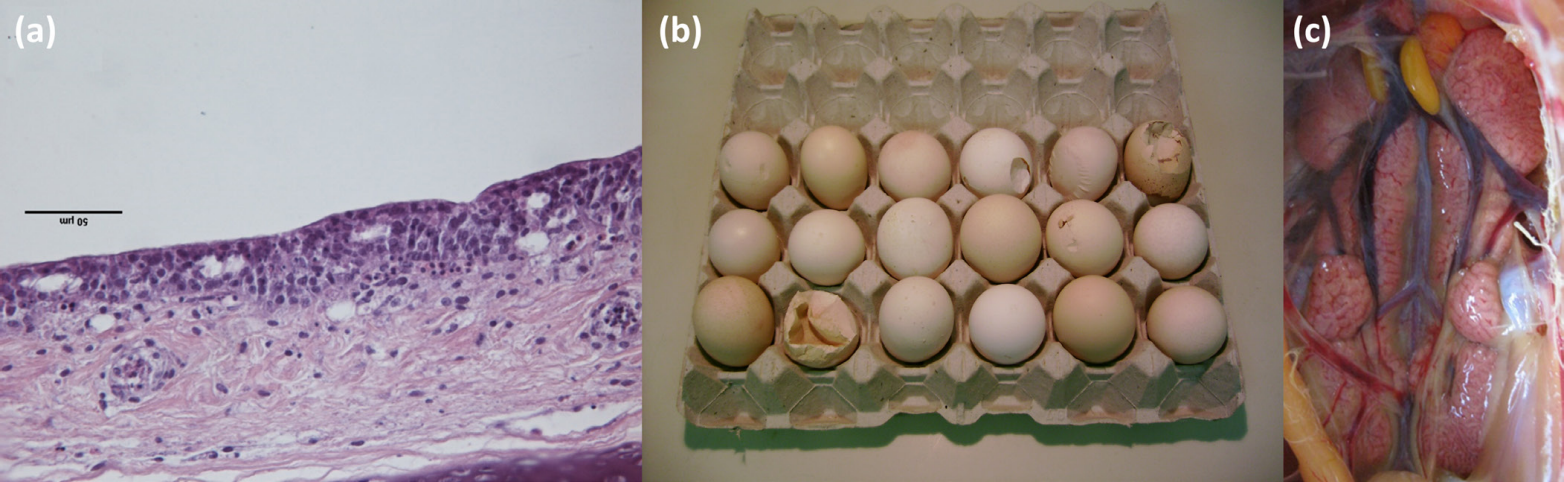
Loss of cilia and lymphocytic infiltration in trachea (**a**), abnormal eggs (**b**) and nephritis (**c**) following IBV infection.

Pathological lesions are not pathognomonic in infected birds. Gross lesions include tracheitis, rhinitis, sinusitis and airsacculitis characterized by serous, catarrhal or caseous exudates. Nephropathogenic infections may result in enlarged swollen and pale kidneys with urates accumulation in ureters. Oviductitis is seen in hens resulting in reduced egg production, abnormal eggs being laid (e.g. misshapen, thin, soft, pale, rough-shelled eggs) and in severe cases oviduct atrophy. Microscopically, the virus affects epithelial cells leading to necrosis of the epithelial layer and cilia loss (Fig. 2). Typically, the inflammatory process is characterized by lymphocytes infiltration with a few heterophils present [4].

### Safety

The IBV is part of the *Coronaviridae* family, which also includes viruses such as SARS-CoV, MERS-CoV and SARS-CoV-2, known for their capability of interspecies transmission [10]. However, there is no direct evidence or well-documented cases of IBV infecting humans [4]. The coronavirus jumping species barriers are notable, but IBV is primarily an avian pathogen, and its impact is significant within the poultry industry. The research and surveillance of such viruses are ongoing to understand their pathobiology and the potential for species jump.

## Treatment and resistance

### Treatment

No specific anti-viral therapies are commercially available. The disease is primarily managed through biosecurity measures, good husbandry practices and vaccination. Vaccination is the primary control strategy designed to protect against specific strains. Several live-attenuated and inactivated vaccines are available. Homologous vaccines confer higher protection towards the identical viral genotypes than heterologous ones [11]. Cross-protection tends to diminish as the degree of amino acid identity in the S1 subunit decreases [12]. Multiple administrations are necessary to provide and maintain protective immunity, particularly against respiratory infections [4]. While inactivated vaccines are injectable, live-attenuated vaccine preparations are generally administered via routes that enable mass vaccination, for example, by spray vaccination, water addition or gel administration at the hatchery. Vaccine-induced protection varies considerably based on the type of vaccine, administration, circulation of natural isolates, infectious dose, and genetic and antigenic variants. Live-attenuated vaccines applied either by spray or in water are by far the most used. During an outbreak, supportive care, such as ensuring optimal environmental conditions and providing good nutrition, can help manage symptoms and reduce stress in the flock, which can help improve recovery rates. Additionally, secondary bacterial infections are common in birds affected by IBV, so antibiotics may be used to treat or prevent these secondary infections, although they do not affect the virus itself [4].

### Resistance

No data about the genetic resistance of commercial lines of chickens to IBV has been reported. Correct vaccination practices induce immunological resistance to infection.

## Pathogenic strategies: host range, host response, transmission, infection and virulence factors

### Host range

Domestic chickens (*Gallus gallus*) are the primary IBV host, however, other Galliformes such as ring-necked pheasants (*Phasianus colchicus*) and red-legged partridges (*Alectoris rufa*) are also commonly infected. Related non-IBV gammacoronaviruses have been isolated from other avian species such as turkeys, teal, geese, pigeons, guinea fowl, ducks and other wild birds. However, these viruses may differ genetically and antigenically from those commonly detected in domestic chickens [13].

### Host response

Various immunological defence mechanisms have been shown to neutralize the virus, including non-specific immune responses. Both innate and adaptive immunity play a role in the induction of the immune response. Humoral immunity can inhibit viral replication and viremia. Cell-mediated immunity is a key immunoregulatory factor following IBV infection and is critical to viral clearance [11]. Local and mucosal immunity is important in preventing respiratory infections, especially in respiratory tissues. Maternally derived antibodies can also provide partial protection *in-ovo* and post-hatch, although they can also interfere with host responses to vaccination [11]. The immune response efficacy depends on its ability to block and neutralize the spike protein of the virus (especially the outer S1 subunit) [11]. There is also evidence that the nucleocapsid protein can prime a protective immune response [14]. Both active (cell-mediated and humoral) and innate immunity play an important role in controlling infections [4].

### Transmission

**Fig. 3.**
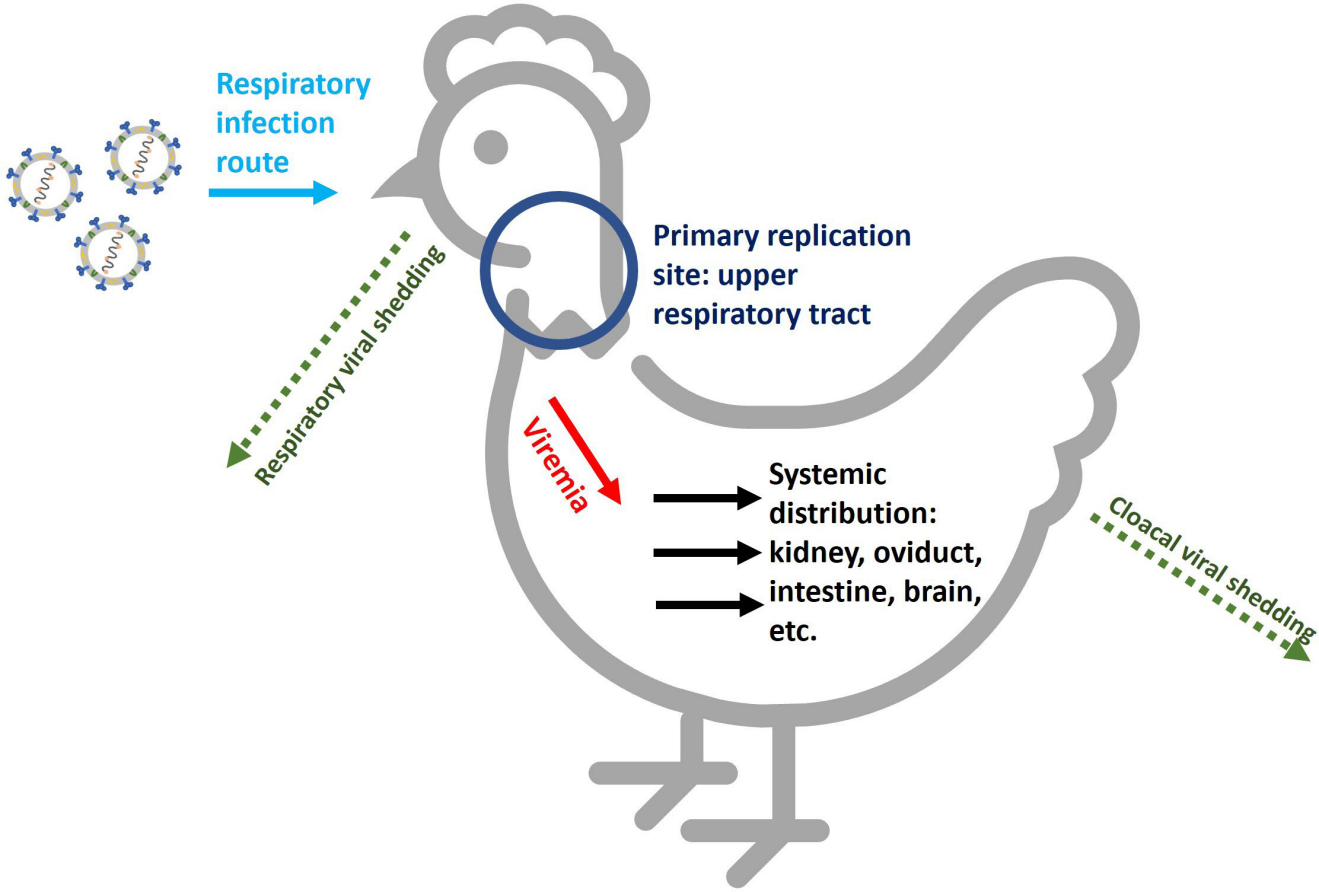
Schematic representation of IBV infection cycle

IBV is a highly contagious virus that spreads rapidly among chickens [15]. Transmission is mainly by inhalation or ingestion of viral particles by direct or indirect contact through aerosol droplets (Fig. 3). The S glycoprotein mediates host-cell attachment, virus and cell membrane fusion, and entry into the host cell. Once in the cell, the viral genome acts as mRNA, initiating viral replication.

The oral-faecal route is spread through the ingestion of faeces-contaminated feed and water. Fomites, namely, contaminated objects, personnel clothing and shoes, can spread the disease [4]. The biosecurity process can help reduce the spread of the disease in poultry premises.

### Virulence factors

The spike protein, like other coronaviruses, is an essential factor with regard to host-cell range and pathogenicity. However, other structural and non-structural proteins have also been implicated in infection outcomes [11], such as the accessory proteins 3a and 3b. Viruses engineered to lack these proteins showed attenuated pathogenicity; and a more significant effect with the loss of 3b [16].

## Epidemiology, prevention and risk groups

### Epidemiology

IBV is present worldwide, and several serotypes and genotypes have been detected in all continents except for Antarctica. Several strains can cocirculate in a given region, some detected in all continents, whereas others seem to have a regional distribution [17].

### Prevention

Good biosecurity is essential for controlling all avian diseases within poultry farms. It involves the implementation of measures to prevent pathogen incursion, such as restricted or controlled site access, providing appropriate personal protective equipment (e.g. boots and uniform), farm equipment and litter sanitation, and foot and wheel baths [18]. In areas with a high density of poultry farms, keeping chickens free from IBV is virtually impossible. Consequently, vaccines are regularly administered as described.

### Risk factors

The density of commercial poultry farms within an area is an important risk factor, mainly where chickens are the main species present. All ages are susceptible, but the disease tends to be more severe in young birds, often causing mortality, especially for nephropathogenic strains. As age increases, chickens become more resistant. However, other environmental factors (ventilation, CO_2_, ammonia presence, etc.) or comorbidity (typically bacterial, e.g. *E. coli*) can have an impact on the infection outcome [4].

### Risk groups

Naive non-vaccinated commercial chickens (both broilers and laying hens) are the most at risk to be infected with a severe impact on meat and egg production [4].

## Open questions

1. Can novel therapeutic approaches, such as antiviral drugs, effectively treat IBV infections in poultry?
2. How can existing vaccines be modified promptly to the evolving IBV serotypes?
3. Can environmental factors and poultry farming practices influence the mutation rate and evolution of IBV?
4. What specific genetic or molecular factors contribute to the virulence and transmissibility of the emerging strains?
5. What lessons can be learnt from the IBV control strategy that can be considered for the mitigation of other coronavirus epidemics?
